# Risk of developing hyperkalemia in patients with hypertension treated with combination antihypertensive therapy – a retrospective register-based study

**DOI:** 10.1038/s41440-024-01894-2

**Published:** 2024-10-31

**Authors:** Fatma Luai Mahdi Al-Janabi, Fatme Moussa, Sarah Taleb, Peter Derek Christian Leutscher, Marc Meller Søndergaard, Dorte Melgaard, Peter Søgaard, Christian Torp-Pedersen, Kristian Kragholm, Maria Lukács Krogager

**Affiliations:** 1 Faculty of Medicine, Aalborg University, Aalborg, Denmark; 2 North Denmark Regional Hospital, Center for Clinical Research, Hjorring, Denmark; 3 Department of Cardiology, Aalborg University Hospital, Aalborg, Denmark; 4 Department of Acute Medicine and Trauma Care, Aalborg University Hospital, Aalborg, Denmark; 5 Department of Cardiology and Clinical Research, Copenhagen University, Nordsjællands Hospital, Hillerød, Denmark

**Keywords:** Beta blockers, Calcium channel blockers, Mineralocorticoid receptor antagonists, Potassium, Renin angiotensin system inhibitors

## Abstract

The risk of hyperkalemia in relation to different combinations of antihypertensive therapy remains to be elucidated. In this Danish register-based study, we aimed to investigate the risk of developing hyperkalemia in relation to different combinations of antihypertensive therapy. Using incidence density matching, we matched a hyperkalemic patient to five normokalemic patients on eGFR groups, age, sex, and time between study entry and date of potassium measurement. Combination therapies were subdivided into eight groups: beta blockers (BB) + calcium channel blockers (CCB), BB + renin angiotensin system inhibitors (RASi), BB + RASi + mineralocorticoid receptor antagonists (MRA), CCB + RASi, CCB + RASi + thiazides, CCB + thiazides, RASi + thiazides, and other combinations. Multivariable conditional logistic regression was used to estimate the odds of hyperkalemia within 90 days for each of the eight antihypertensive combination therapies. A total of 793 patients with hyperkalemia were matched to 3598 normokalemic patients. In multivariable analysis, odds of developing hyperkalemia when being treated with BB + RASi + MRA was 1.95 (95% CI, 1.39–2.72) compared to RASi + thiazides (reference). CCB + thiazides (OR, 0.76 [95% CI, 0.45–1.28]) and CCB + RASi + Thiazid (OR 0.81 [95% CI, 0.51–1.28]) were among the others not significantly associated with hyperkalemia. Combinations of BB + RASi + MRA were significantly associated with an increased risk of developing hyperkalemia within 90 days of initiating treatment.

**Figure:**
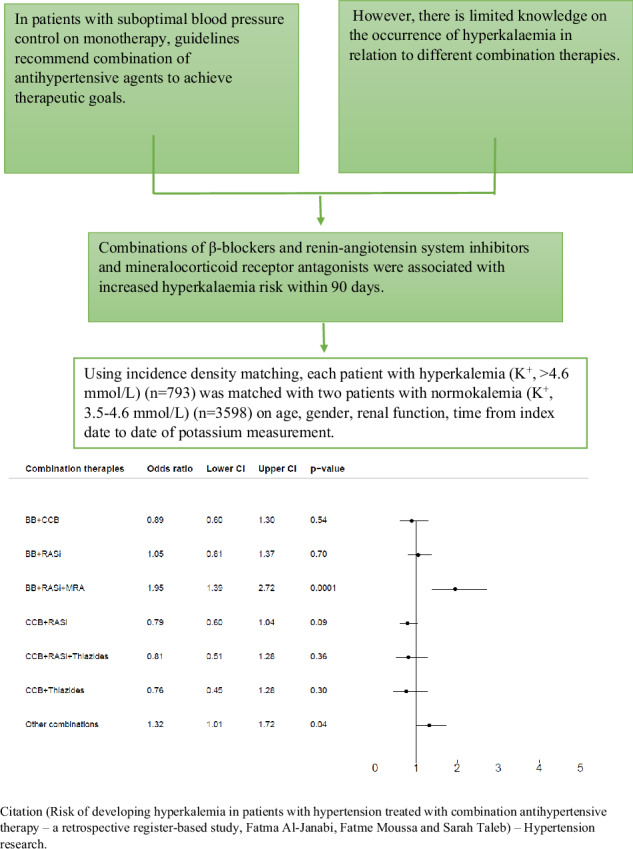
Patients treated with BB + RASi + MRA within 90 days of treatment initiation, were associated with an increased hyperkalemia risk. When treating hypertensive patients with combination antihypertensive therapy, identifying and monitoring patients with a high risk of dyskalemias is a crucial goal to avoid serious adverse effects and detrimental outcomes related to dyskalemia.

Patients treated with BB + RASi + MRA within 90 days of treatment initiation, were associated with an increased hyperkalemia risk. When treating hypertensive patients with combination antihypertensive therapy, identifying and monitoring patients with a high risk of dyskalemias is a crucial goal to avoid serious adverse effects and detrimental outcomes related to dyskalemia.

## Introduction

Combination antihypertensive therapy, i.e., two or more pharmaceutical drugs, is commonly used in the treatment of hypertension. Calcium channel blockers (CCB), angiotensin receptor blockers (ARBs), angiotensin-converting enzyme inhibitors (ACEIs), β-blockers (BB), and thiazides/thiazide-like diuretics are the five main drug classes recommended by the current European guidelines for the management of hypertension [1]. If the patient does not have an optimal response with monotherapy, guidelines recommend supplementing with other antihypertensive drugs until target blood pressure is reached [1]. Treatment with antihypertensive drugs may influence potassium homeostasis, mainly ACEIs, ARBs, and diuretics through previously described mechanisms [2]. Preventing potassium imbalances in patients with hypertension is important, as they can lead to hospitalization, development of malignant arrhythmias, or sudden cardiac death [2–5]. A previous study concluded that potassium levels outside the interval of 4.1–4.7 mmol/L were associated with increased mortality risk in patients with hypertension [6]. Hyperkalemia was defined as potassium >4.6 mmol/L for two reasons. First, results from the Nordic cohort cohort showed separate reference intervals for serum and plasma potassium and were concluded to be almost identical. According to the Nordic cohort hyperkalemia was also >4.6 in serum [7]. Second, in Denmark the laboratory data also marked potassium values as abnormal when >4.6 mmol/L. In patients with heart failure, it is well known that both medication such as ACEIs and cardiorenal syndrome can lead to hyperkalemia. However, few studies have investigated the risk of hyperkalemia in patients with hypertension treated with combination therapy. By using the Danish administrative registries, we investigated the risk of developing hyperkalemia within 90 days after initiation of different antihypertensive therapy combinations.

## Methods

### Databases

In Denmark, all residents are given a 10-digit personal and unique social security number that is used in all healthcare and governmental contacts, allowing linkage of data across national administrative registries. The Danish Civil Registration System [8] was used to collect data about gender and age. Information about discharge diagnoses, dates of operation, procedure codes, hospital admission and discharge dates were obtained from the Danish National Patient Registry [9]. The ICD version 10 has been in use in Denmark since 1994 [10]. The Danish National Prescription Registry has been covering and collecting information on each individual’s drug redemption including all prescriptions given from the Danish pharmacies since 1995 [11]. Pharmacies in Denmark are required by law to register filled drug prescriptions in The Danish National Prescription Registry. Laboratory data used in this study were available from four of five regions in Denmark: the North Denmark Region, Region of Southern Denmark, Region Zealand, and the Capital Region of Denmark. The data covers approximately 4,000,000 inhabitants.

### Study design and population

In this retrospective, register-based, nested, case-control study, hypertension was defined as prescription for a minimum of two antihypertensive drugs in two consecutive yearly quarters.

Patients were included in this study in the second quarter and this date is referred to as the index date. The Anatomical Therapeutic Chemical (ATC) codes of the antihypertensive drugs used to define patients with hypertension are listed in Supplementary Table 1.

The definition of hypertension we used has previously been validated in a study by Olesen et al, where the authors found a specificity of 94.7% and a positive predictive value of 80% [12, 13].

The window period of 90-days for retaining the first potassium measurement was considered appropriate as a review showed that, in patients with hypertension, dyskalemias as a result of adverse drug reaction peaked at 3 months [14]. A bar chart was constructed to illustrate time to first potassium measurement after initiating combination antihypertensive therapy (Supplementary Fig. 1).

Further inclusion criteria were 1) age 18 years or above, and 2) a corresponding first potassium measurement within 90 days of initiating combination therapy. Exclusion criteria were 1) patients without potassium measurement up to 30 days before index date, 2) hypokalemia or hyperkalemia up to 30 days before index date, 3) hypokalemia at the first potassium measurement after combination therapy initiation, 4) patients diagnosed with SIADH, diabetes insipidus, hyperaldosteronism, and/or Addison, and 5) prescription of loop diuretics as they are neither one of the five main drug classes as first line treatment [15] nor commonly used in treatment of heart failure [16].

The retrospective, register-based, nested, case-control study used incidence density matching, where we matched each patient with hyperkalemia (K^+^, >4.6 mmol/L) to five patients with normokalemia (K^+^, 3.6–4.6 mmol/L) [7]. The two groups were matched on renal function according to eGFR estimated at baseline, age, sex, and time between index date and potassium measurement.

During the study period 1995–2018, there was a switch from measuring potassium in serum to plasma in Danish hospital laboratories. Blood tests, including blood tests from the primary care setting, were analyzed in these laboratories. The switch did not occur simultaneously in all clinics and hospitals. The normal ranges for serum and plasma blood potassium concentrations do not differ substantially [7]. This study referred to all measurements as serum potassium [13]. The population flow chart with inclusion and exclusion criteria is shown in Supplementary Fig. 2.

### Exposure

The independent variable defining the different combinations of antihypertensives was coded as a categorical variable with eight groups:

1. BB + CCB
2. BB + RASi
3. BB + RASi + MRAs
4. CCB + RASi
5. CCB + RASi + thiazides
6. CCB + thiazides
7. RASi + thiazides (reference)
8. Other combinations

Groups 1, 4, 5, and 6 consisted of combinations of CCB with other blood pressure drugs. These groups contain one type of CCB, which is dihydropyridine derivatives, such as amlodipine, listed in the Supplementary Table 1.

### Comorbidities, procedures, and concomitant medication

To characterize the population, we identified the following comorbidities up to five years before the index date: acute myocardial infarction, heart failure, ventricular tachycardia or fibrillation, atrial flutter or fibrillation, ischemic heart disease, chronic obstructive pulmonary disease, malignancy, hemodialysis, and diabetes mellitus. Supplementary Table 2 contains the diagnosis codes used to identify the specific comorbidities and/or concomitant medication.

The chronic kidney disease epidemiology collaboration (CKD-EPI) [17] formula was used to calculate the estimated glomerular filtration rate (eGFR), in order to assess each patient’s renal function. eGFR was divided into six groups according to KDIGO guidelines [18]. Resulting in the following six groups:

1) Normal = ≥ 90

2) Mildly decreased = 60–89

3) Mildly to moderately decreased = 45–59

4) Moderately to severely decreased = 30–44

5) Severely decreased = 15–29

6) Kidney failure = <15

Cases were matched with each respective eGFR group in addition to age, sex, and time between study entry and date of potassium measurement. Serum creatinine is part of the formula and was obtained the same day as potassium measurement. If patients had no creatinine measurement the same day, the measurement closest, within a week, to the potassium measurement was chosen. If no creatinine measurements were available the same day as potassium or within a week from potassium measurement, patients were excluded.

The following drugs were identified from the Danish National Prescription Registry, as they are commonly linked to a disturbance in potassium levels: β2-agonists, potassium supplements, laxatives, antimicrobials, macrolides, mineralo- and glucocorticoids, and xanthines [11]. Potassium supplements were either as a single pill therapy in combination with an antihypertensive (Anatomic Therapeutic Chemical C03) or an individual pill (ATC A12B).

### Statistical analysis

Categorical variables are presented as counts and percentages, and continuous variables as medians with 25th to 75th percentiles. Differences between continuous variables were compared using the Kruskal Wallis tests, and χ2 was used for categorical variables.

For patients who had available potassium measurements within 90 days from index date and no potassium imbalances up to 30 days before index date, cumulative incidence curves were estimated using Aalen-Johansen estimators.

**Fig. 1:**
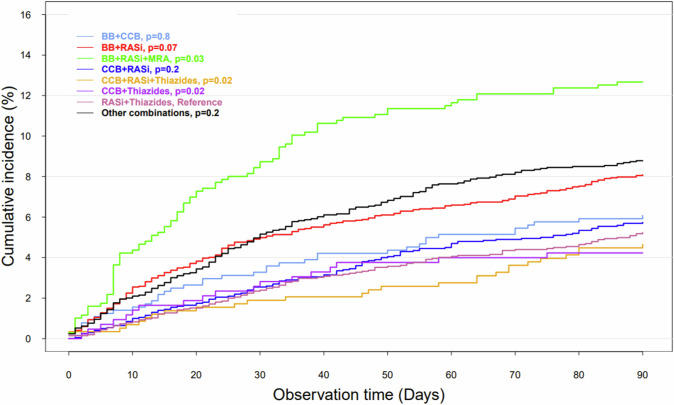
Cumulative incidence proportion curve of hyperkalemia in patients treated with combination antihypertensive therapy. Cumulative incidence proportion of hyperkalemia in patients treated with combination antihypertensive therapy who had available potassium measurements within 90 days from index date and no potassium imbalances up to 30 days before index date (*n* = 11.152)

We used conditional logistic regression analysis to analyze the association between the different combination antihypertensive therapies and the risk of developing hyperkalemia within 90 days from treatment initiation. In the regression model, group 7 (RASi + thiazides) was used as reference for three reasons. First, it is one of the most redeemed combinations. Second, it has one of the lowest cumulative incidences of hyperkalemia (Fig. 1). Third, thiazides diuretics are associated with hypokalemia, whereas RASi are associated with hyperkalemia. By using this combination, we would expect that the dyskalemia risk is reduced [13, 19].

The multivariable model was adjusted for initial serum sodium, potassium supplements, NSAIDs, and macrolides.

A two-sided *P* value < 0.05 was considered statistically significant.

SAS, version 9.4 (SAS Institute, Inc, Cary, NC) and R, version 4.1.3 (R Core Team [2018]) were used for analyses and data management [20].

### Ethics

Informed consent or ethical approval is not needed for register-based studies in Denmark. Use of the data sources for this study is authorized by a data responsible institute in the Capital Region of Denmark (approval number P-2019-404) in compliance with the General Data Protection Regulation (GDPR).

## Results

When matching on age, sex, eGFR divided into six different groups, and time from index date to potassium measurement, 3598 controls and 793 cases were found. Out of the eight combination antihypertensive therapies, the most common were: BB + RASi (24.7%), and CCB + RASi (20.0%). A higher incidence of BB + RASi (27.2%) and CCB + RASi (14.5%) was observed in patients with hyperkalemia compared to the other groups. Out of the 793 cases with hyperkalemia, 34.9% were treated with potassium supplement (Table 1).

**Table 1:** Demographic and clinical characteristics of the matched population

	K+ <4.6 mmol/L (*n* = 3598)	K+ >4.6 mmol/L (*n* = 793)	Total (*n* = 4391)	*p* value
*Gender*^a^	1460 (40.6)	327 (41.2)	1787 (40.7)	
Female				0.76
Age, median (range)^a^	71 (19, 98)	71 (19, 98)	71 (19, 98)	0.80
Days from hypertension to potassium measurement, median (range)^a^	27 (0, 90)	26 (0, 90)	26 (0, 90)	0.81
*Renal function*^a^				
eGFR >90 mL/min/1.73 m^2^	636 (17.7)	134 (16.9)	770 (17.5)	
eGFR 89–60 mL/min/1.73 m^2^	1715 (47.7)	350 (44.1)	2065 (47.0)	
eGFR 59–45 mL/min/1.73 m^2^	786 (21.8)	164 (20.7)	950 (21.6)	
eGFR 44–30 mL/min/1.73 m^2^	359 (10.0)	90 (11.3)	449 (10.2)	
eGFR 29–15 mL/min/1.73 m^2^	92 (2.6)	41 (5.2)	133 (3.0)	
eGFR<15 mL/min/1.73 m^2^	10 (0.3)	14 (1.8)	24 (0.5)	<0.0001
*Treatment combinations*				
BB+CCB	227 (6.3)	39 (4.9)	266 (6.1)	
BB+RASi	868 (24.1)	216 (27.2)	1084 (24.7)	
BB+RASi+MRA	242 (6.7)	87 (11.0)	329 (7.5)	
CCB+RASi	763 (21.2)	115 (14.5)	878 (20.0)	
CCB+RASi+thiazid	165 (4.6)	27 (3.4)	192 (4.4)	
CCB+thiazid	121 (3.4)	18 (2.3)	139 (3.2)	
RASi+thiazid	571 (15.9)	107 (13.5)	678 (15.4)	
Other combinations	641 (17.8)	184 (23.2)	825 (18.8)	<0.0001
*Individual antihypertensive drug*				
BB	1812 (50.4)	468 (59.0)	2280 (51.9)	<0.0001
CCB	1559 (43.3)	258 (32.5)	1817 (41.4)	<0.0001
RASi	3023 (84)	658 (83)	3681 (83.8)	0.50
MRA	445 (12.4)	164 (20.7)	609 (13.9)	<0.0001
Diuretic	1162 (32.3)	241 (30.4)	1403 (32.0)	0.32
Thiazides	1147 (31.9)	237 (29.9)	1384 (31.5)	0.29
*Comorbidities*				
Heart failure	336 (9.3)	194 (24.5)	530 (12.1)	<0.0001
IHD/MI	454 (12.6)	207 (26.1)	661 (15.1)	<0.0001
VT/VF	112 (3.1)	40 (5.0)	152 (3.5)	0.01
Atrial flutter/fibrillation	328 (9.1)	132 (16.6)	460 (10.5)	<0.0001
COPD	171 (4.8)	61 (7.7)	232 (5.3)	0.001
Hemodialysis	6 (0.2)	6 (0.8)	12 (0.3)	0.01
Diabetes	513 (14.3)	147 (18.5)	660 (15.0)	0.003
Malignancy	506 (14.1)	130 (16.4)	636 (14.5)	0.10
NSAIDs	7 (0.2)	8 (1.0)	15 (0.3)	0.001
*Other*				
Serum sodium, median (range)	139 (114, 156)	139 (119, 150)	139 (114, 156)	0.41
Potassium supplement	1192 (33.1)	277 (34.9)	1469 (33.5)	0.35
Potassium chloride (single pill combination with an antihypertensive drug)	767 (21.3)	166 (20.9)	933 (21.2)	0.85
Potassium chloride	501 (13.9)	132 (16.6)	633 (14.4)	0.05

We attribute ≤3 to variables with values between 1 and 3 to secure anonymity and protection of personal data

*CCB* calcium channel blockers, *BB* β-blockers, *COPD* chronic obstructive pulmonary disease, *eGFR* estimated glomerular filtration rate, *ICD* implantable cardioverter defibrillator, *IHD/MI* ischemic heart disease/myocardial infarction, *MRA* mineral receptor antagonist, *RASi* renin–angiotensin system inhibitors, *VT/VF* ventricular tachycardia/ventricular fibrillation

^a^Represents the variables we matched on

Population characteristics, from where controls and cases were identified, are illustrated in Supplementary Table 3. As seen in Supplementary Fig. 2, the population flowchart, 11,152 patients with hypertension had a potassium measurement within 90 days of index date during the period from 1995 to 2023. A total of 7.1% (793) of these patients had blood potassium concentrations over 4.6–mmol/L. Additionally, 34.3% of the 11,152 redeemed potassium supplements. Of these, 23.5% represented potassium chloride as a single pill combined with a drug for hypertension.

### Antihypertensive combination therapies and rate of hyperkalemia

The cumulative incidence proportion of hyperkalemia in patients treated with combination antihypertensive therapy is shown in Fig. 1. Patients treated with RASi + thiazides had the lowest incidence of hyperkalemia, whereas patients treated with BB + RASi + MRA had a significantly higher incidence of hyperkalemia compared to the other groups (*P* = 0.03).

**Fig. 2:**
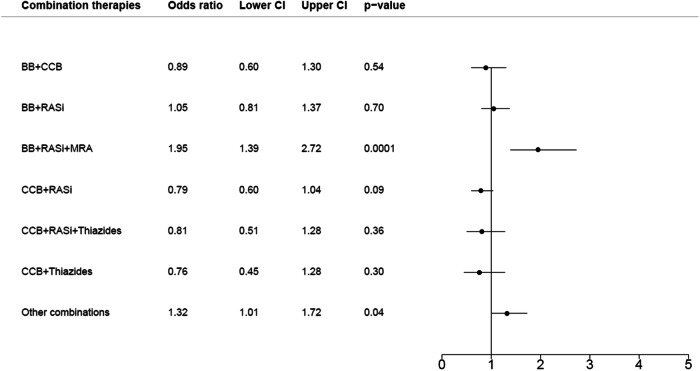
Forest plot made of a multivariable conditional logistic regression analysis regarding the hyperkalemia development. The population was matched on age, renal insufficiency, sex, and time between the index date to the first potassium measurement. The model was adjusted for serum sodium, potassium supplements, NSAIDs, and macrolides. The combination of renin-angiotensin system inhibitors with thiazides was used as reference. BB defines β-blockers, MRA mineral receptor antagonist, RASi renin-angiotensin system inhibitors, CCB calcium channel blockers

The adjusted conditional logistic regression analysis (Fig. 2), with RASi + thiazides as reference showed an OR of 1.95 (95% CI, 1.39–2.72) for development of hyperkalemia in patients prescribed with BB + RASi + MRA. Following drug combination without increased hyperkalemia risk were: CCB + thiazides (OR, 0.76 [95% CI, 0.45–1.28]), CCB + RASi + Thiazid (OR 0.81 [95% CI, 0.51–1.28]), CCB + RASi (OR, 0.79 [95% CI, 0.60–1.04]), BB + CCB (OR, 0.89 [95% CI, 0.60–1.30]), and BB + RASi (OR 1.05 [95% CI, 0.81–1.37]).

The univariable analysis showed similar results, however combination BB + RASi also showed a statistically significant association with the development of hyperkalemia with (OR, 1.57 [95% CI, 1.22–2.01]) within 90 days of initiating treatment (Supplementary Fig. 3).

Supplementary Tabel 4 shows the distribution of concomitant antihypertensive therapy among patients with hyperkalemia administered MRA. We observed that 79.3% of the patients redeemed RASi and 39% potassium supplementation.

## Discussion

This study found hyperkalemia among patients treated with combination antihypertensive therapies was common, and the antihypertensive drug combinations with the highest odds of developing hyperkalemia was BB + RASi + MRA.

Current guidelines recommend combination antihypertensive drug treatment when patients do not achieve targeted blood pressure using monotherapy [1]. While it is important to regulate elevated blood pressure to prevent and/or manage cardiovascular disease, combination therapy affects potassium homeostasis. In our study, 7.1% of the patients developed hyperkalemia within 90 days from combination therapy initiation. A large Swedish study investigated determinants of hyperkalemia and hypokalemia and documented that patients with hypertension had 1.80 and 1.05 times higher odds of developing, hyper- and hypokalemia, respectively, within three years. Furthermore, the study observed increased risk of dyskalemia in the presence of RASi and diuretics [21]. This aligns with our findings where the use of BB + RASi + MRA, and BB + RASi, increased the odds of hyperkalemia. However, the two studies are not directly comparable as the definition of hypertension and study aims were different. Making direct comparisons is difficult as no other known studies have investigated the association between hyperkalemia and the same antihypertensive combination therapies as has this study. Due to the lack of studies investigating the risk of hyperkalemia associated with these specific combinations, we searched literature on each antihypertensive drug individually.

### BB and RASi

The majority of current literature compares the risk of developing hyperkalemia in patients treated with RASi as monotherapy for hypertension with patients on different monotherapies, such as CCB, BB, and thiazides [22, 23]. A systematic review finds an association between treatment with RASi and hyperkalemia. Although the study focuses on patients with chronic heart failure, whereas our study focuses on hypertension with comorbidities such as heart failure, it also mentions that patients with advanced stages of chronic kidney disease, diabetes, and resistant hypertension have a higher risk of developing hyperkalemia [19].

Current guidelines allow any of the major antihypertensive drug classes (angiotensin-converting enzyme inhibitors, angiotensin receptor blockers, calcium channel blockers, beta-blockers, diuretics) to be considered for initiation combination [24]. BBs are highly used in our population together with RASi (83%) and CCBs (40%). This is not uncommon in a population treated with combination antihypertensive therapy. When looking at the burden of different comorbidities that also can be treated with BBs, we observed that the distribution of atrial fibrillation, heart failure and ischemic heart disease lies between 11.5% and 18.3% (Supplementary Table 3). A study showed high use of betablockers as monotherapy, when treating hypertension, and as prevention consistent with guidelines for secondary cardiovascular disease. Among patients with cardiovascular disease, treatment with ACEI or ARB or a betablocker are commonly used for secondary prevention of cardiovasculardisease [25].

Studies have shown that beta-blockers, mostly non-selective, can generate the development of hyperkalemia. This can happen through two different mechanisms. Beta blockers can decrease the activity of the sodium potassium pump and thereby interact with the cellular uptake of potassium. Another mechanism can be described by the indirect blockage of renin release through its direct inhibiting effects on the sympathetic nervous system [24, 25]. A case report study concludes that hyperkalemia induced by beta-adrenergic receptor blockade can occur in 1–5% of patients, and further indicates a higher risk of it occurring in non-cardio-selective BB than in cardio-selective BB [26]. Yet this study, unlike ours, investigates hypertensive patients treated with BB with comorbidities such as renal dysfunction and/or insulin insufficiency and, based on this fact, concludes that BB are associated with hyperkalemia.

### BB + RASi + MRA

No previous studies have reported increased hyperkalemia risk in patients treated with BB + RASi + MRA. We found that the use of BB is associated with increased hyperkalemia risk, especially in patients with hypertension and concomitant renal dysfunction and insulin insufficiency, while use of RASi such as MRA, ACEIs, and ARBs is associated with increased hyperkalemia [27–29]. A systematic review and meta-analysis investigated the antihypertensive effects of MRA + RASi combination therapy in patients with hypertension and DM. It found that combining MRA and RASi induced a significant reduction in SBP and DBP, a reduction in urinary albumin excretion, and a slight elevation in blood serum potassium concentrations [30]. This aligns with our findings that BB + RASi + MRA are associated with hyperkalemia.

The mechanisms through which RASi cause hyperkalemia are principally similar, through their influence on the production or activity of aldosterone. ACEIs and ARBs reduce aldosterone secretion and thereby limit urinary excretion of potassium, predisposing to hyperkalemia. In contrast, MRAs increase the resistance to aldosterone by blocking the interaction between aldosterone and the mineralocorticoid receptors [28].

As observed in Supplementary Table 4 among patients with hyperkalemia administered MRA a great majority of patients were prescribed RASi and potassium supplementation as well. All three drugs are known to induce hyperkalemia [24, 31]. This combination is not common for the general hypertension population, yet it can be used in resistant hypertension or heart failure [32].

### Risk of electrolyte abnormalities due to antihypertensive drugs

Antihypertensive drugs are not only associated with the risk of developing hyperkalemia. They are also associated with a general risk of electrolyte abnormalities, such as hypokalemia and hyponatremia. Hypokalemia can be a serious condition as it can lead to muscle weakness and lethal arrhythmias. Hyponatremia is known to increase the risk of neurological abnormalities such as impaired consciousness [4, 13, 33, 34]. This study found that patients treated with RASi + thiazides had the lowest incidence of hyperkalemia. These results align with a study that finds an association between the development of hypokalemia and the treatment with antihypertensive combination therapy with thiazide diuretics and CCB, RASi or BB [13]. Regarding hyponatremia, ACE and ARBs can reduce the ability to regulate natrium and water balance in the body [35]. Additionally hyponatremia is more frequent in patients treated with thiazide diuretics [36]. Another study showed an association between the use of CCBs, ACE, BB and ARB and an increased risk of hyponatremia as the cause of hospitalization [37].

### Limitations

Overdose, compliance issues, and other nondifferential misclassification could influence the results achieved in this study. Unmeasured confounders such as diet, dehydration, vomiting, diarrhea, and blood loss could also interfere with disruption of potassium homeostasis, without having the possibility to account for them through the administrative registries. The clinical indication for blood tests, symptoms of potassium imbalances, electrocardiographic changes, or blood tests was unavailable. According to current guidelines, hypertensive patients are recommended to have standard blood tests taken and their blood pressure monitored within the first two to three months from initiating treatment. Thus, we believe that cases where our population has been specifically tested for dyskalemia are limited.

Besides antihypertensive medication, the causes of hyperkalemia are numerous and include metabolic acidosis and dehydration. In order to investigate an association between different combinations of antihypertensive drugs and hyperkalemia that is as unbiased/unconfounded as possible, we required that patients included in this study were normokalemic up to 30 days from the index date.

Hypertension was defined as taking at least two classes of antihypertensive drugs over two consecutive quarters.This approach has its limitations despite being previously validated [11]: First, by identifying patients with hypertension based on antihypertensive medication, we do not have the precise indication for starting this treatment. This can lead to misclassification bias, as we could have identified patients with other cardiovascular conditions such as heart failure, atrial fibrillation or ischemic heart disease. However, several studies have shown that cardiovascular disease is predominantly caused by a rightward shift in the distribution of blood pressure [38].

Second, by using the current definition of hypertension, patients treated with monotherapy were excluded. Misclassification bias would have been increased if patients were identified solely by monotherapy as the indication for treatment was not available.

The analyses were performed on predefined antihypertensive drug combinations based on their distribution. Analysis of other combinations would be difficult both to perform, but also to interpret or present because of the low distribution, in some cases microdata.

A sensitivity analysis using higher criteria of serum potassium levels was not optimal to perform in this study, as there were only 23 with potassium measurements >5.5 mmol/.

We tried to perform a sensitivity analysis using a higher cut off value for the definition of hyperkalemia (>5.5 mmol/L). Only 23 patients had potassium values above 5.5 mmol/L, which makes both interpretation and generalizability difficult.

## Conclusions

Combinations of BB + RASi + MRA within 90 days of treatment initiation, were strongly associated with an increased risk of hyperkalemia, regardless of potassium supplements.

## Perspectives

The treatment of hypertension in clinical practice could be optimized, as our findings and several studies indicate that hyperkalemia is a common side effect of combination antihypertensive therapy. It is crucial to identify patients at risk of dyskalemias to focus on addressing risk factors associated with potassium disturbances. This is, for instance, emphasized in this study, where patients treated with BB + RASi + MRA had an increased probability of developing hyperkalemia within 90 days of the index date, regardless of potassium supplements. Thus, awareness when identifying and monitoring patients at high risk of dyskalemias is a crucial goal to potentially avoid serious adverse effects and detrimental outcomes related to dyskalemia. However, CCB + thiazides is associated with the lowest risk of developing hyperkalemia, which suggest that physicians should consider a combination of antihypertensives with the opposite effect on potassium homeostasis.

## Supplementary information

Supplementary Table 1

Supplementary Table 2

Supplementary Table 3

Supplementary Table 4

Supplementary Figure 1

Supplementary Figure 2

Supplementary Figure 3

## Data Availability

Statistics Denmark is the official and central authority for Danish statistics that holds the data used in this study. Due to restrictions related to Danish law and to protect patient privacy, the combined set of data used in this study can only be made available through a trusted third party, Statistics Denmark. A university-based Danish scientific organization can be authorized through Statistics Denmark. When organizations are authorized, it is possible for scientists in and out of Denmark to get access through the organization. Authorized scientists can by request get access to data through contacting Statistics Denmark.
