# Supplementary material for: Risk of developing hyperkalemia in patients with hypertension treated with combination antihypertensive therapy – a retrospective register-based study
Source: Hypertens Res. 2024 Oct 31;48(1):378–87. doi: 10.1038/s41440-024-01894-2 (PMC11700848; doi:10.1038/s41440-024-01894-2)
Supplement: Supplementary file 1 — Supplementary Table 1 [file 41440_2024_1894_MOESM1_ESM.docx]

| **ATC codes** | **Name of drug** |
| --- | --- |
| C02A | Antiadrenergic agents, centrally acting |
| C02B | Antiadrenergic agents, ganglion blocking |
| C02C | Antiadrenergic agents, peripherally acting |
| C03AA | Thiazides |
| C03AB | Thiazides and potassium in combination |
| C03BA | Sulfonamides |
| C03BB | Sulfonamides and potassium in combination |
| C03AX | Hydrochlorothiazide |
| C03C | Loop diuretics |
| C03DA | Aldosteron antagonists |
| C03DB | Other potassium sparing agents |
| C03EA | Low-ceiling diuretics and potassium sparing agents |
| C03EB | High-ceiling diuretics and potassium sparing agents |
| C03X | Other diuretics |
| C07A | Beta-blockers |
| C07BA | Beta blocking, non-selective, and thiazides |
| C07BB | Beta blocking, selective, and thiazides |
| C07BG | Alpha and beta blocking and thiazides |
| C07FB | Beta-blockers and calcium antagonists |
| C07E | Beta blocking and calcium antagonist |
| C07FX | Beta-blockers and other combinations |
| C08C | Selective calcium antagonists primarily with vascular effect |
| C08D | Selective calcium antagonists with direct cardiac effect |
| C08E | Non-selective calcium antagonists |
| C08G | Calcium antagonists and diuretics |
| C09AA | Angiotensin converting enzyme inhibitors |
| C09BA | Angiotensin converting enzyme inhibitors and diuretics |
| C09BB | Angiotensin converting enzyme inhibitors and calcium antagonists |
| C09CA | Angiotensin II antagonists |
| C09DA | Angiotensin II antagonists and diuretics |
| C09DB | Angiotensin II antagonists and calcium antagonists |
| C09XA | Renin-inhibitors |
