# Supplementary material for: Risk of developing hyperkalemia in patients with hypertension treated with combination antihypertensive therapy – a retrospective register-based study
Source: Hypertens Res. 2024 Oct 31;48(1):378–87. doi: 10.1038/s41440-024-01894-2 (PMC11700848; doi:10.1038/s41440-024-01894-2)
Supplement: Supplementary file 2 — Supplementary Table 2 [file 41440_2024_1894_MOESM2_ESM.docx]

| **Comorbidities and procedures** | **ICD-10**  **codes** | **Time**  **prior to index**  **date** | **NCSP**  **codes** | **Time**  **prior to index**  **date** | **ATC**  **codes** | **Time**  **prior to index**  **date** |
| --- | --- | --- | --- | --- | --- | --- |
| Ischemic heart disease including myocardial  infarction^1^ | I20-25 | 5 years | KFNG,  KFNA-E | 5 years | − | − |
| Hemodialysis | − | − | BJFD20 | 5 years | − | − |
| Atrial flutter or fibrillation | I48 | 5 years | − | − | − | − |
| Ventricular  tachycardia or fibrillation | I47.2, I49 | 5 years | − | − | − | − |
| Chronic obstructive pulmonary disease | J40-44 | 5 years | − | − | − | − |
| Diabetes | E10-14 | 5 years | − | − | − | − |
| Cancer | C00-99 | 5 years | − | − | − | − |
| Heart failure | I110,  I130,  I132, I42, I50, J81 | 5 years | − | − | − | − |
| **Concomitant**  **medications** | **ICD-10**  **codes** | **Time**  **prior to index**  **date** | **NCSP**  **codes** | **Time**  **prior to index**  **date** | **ATC**  **codes** | **Time**  **prior to index**  **date** |
| Potassium  supplements | − | − | − | − | A12B  C03AB  C03BB  C03CB | 90 days |
| Non-steroidal anti inflammatory drugs | − | − | − | − | M01A | 180 days |
| Macrolides | − | − | − | − | J01FA | 30 days |
