# Supplementary material for: Risk of developing hyperkalemia in patients with hypertension treated with combination antihypertensive therapy – a retrospective register-based study
Source: Hypertens Res. 2024 Oct 31;48(1):378–87. doi: 10.1038/s41440-024-01894-2 (PMC11700848; doi:10.1038/s41440-024-01894-2)
Supplement: Supplementary file 3 — Supplementary Table 3 [file 41440_2024_1894_MOESM3_ESM.docx]

|  | K+ <4.6 mmol/L (n=10359) | K+ >4.6 mmol/L (n=793) | Total (n=11152) | p-value |
| --- | --- | --- | --- | --- |
| Gender |  |  |  |  |
| Female | 4,593 (44.3) | 327 (41.2) | 4,920 (44.1) | 0.10 |
| Age, median (range) | 67( 19, 101) | 71(19, 98) | 67( 19, 101) | < 0.0001 |
| Days from hypertension to potassium  measurement, median (range) | 25( 0, 90) | 26( 0, 90) | 25( 0, 90) | 0.64 |
| Renal function |  |  |  |  |
| eGFR >90 ml/min/1,73m2 | 2,981 (28.8) | 134 (16.9) | 3,115 (27.9) |  |
| eGFR 89-60 ml/min/1,73m2 | 5339 (51.5) | 350 (44.1) | 5,689 (51.0) |  |
| eGFR 59-45 ml/min/1,73m2 | 1371 (13.2) | 164 (20.7) | 1,535 (13.8) |  |
| eGFR 44-30 ml/min/1,73m2 | 515 (5.0) | 90 (11.3) | 605 (5.4) |  |
| eGFR 29-15 ml/min/1,73m2 | 123 (1.2) | 41 (5.2) | 164 (1.5) |  |
| eGFR<15 ml/min/1,73m2 | 30 (0.3) | 14 (1.8) | 44 (0.4) | < 0.0001 |
| Treatment combinations |  |  |  |  |
| BB+CCB | 603 (5.8) | 39 (4.9) | 642 (5.8) |  |
| BB+RASi | 2454 (23.7) | 216 (27.2) | 2,670 (23.9) |  |
| BB+RASi+MRA | 600 (5.8) | 87 (11.0) | 687 (6.2) |  |
| CCB+RASi | 1888 (18.2) | 115 (14.5) | 2,003 (18.0) |  |
| CCB+RASi+Thiazid | 554 (5.3) | 27 (3.4) | 581 (5.2) |  |
| CCB+Thiazid | 408 (3.9) | 18 (2.3) | 426 (3.8) |  |
| RASi+Thiazid | 1941 (18.7) | 107 (13.5) | 2,048 (18.4) |  |
| Other combinations | 1911 (18.4) | 184 (23.2) | 2,095 (18.8) | < 0.0001 |
| Individual antihypertensive drug |  |  |  |  |
| BB | 5093 (49.2) | 468 (59.0) | 5,561 (49.9) | < 0.0001 |
| CCB | 4265 (41.2) | 258 (32.5) | 4,523 (40.6) | < 0.0001 |
| RASi | 8622 (83.2) | 658 (83.0) | 9,280 (83.2) | 0.89 |
| MRA | 1140 (11.0) | 164 (20.7) | 1,304 (11.7) | < 0.0001 |
| Diuretics | 3957 (38.2) | 241 (30.4) | 4,198 (37.6) | < 0.0001 |
| Thiazides | 3902 (37.7) | 237 (29.9) | 4,139 (37.1) | < 0.0001 |
| Comorbidities |  |  |  |  |
| Heart failure | 1324 (12.8) | 194 (24.5) | 1,518 (13.6) | < 0.0001 |
| IHD/MI | 1830 (17.7) | 207 (26.1) | 2,037 (18.3) | < 0.0001 |
| VT/VF | 323 (3.1) | 40 (5.0) | 363 (3.3) | 0.004 |
| Atrial flutter/fibrillation | 1149 (11.1) | 132 (16.6) | 1,281 (11.5) | < 0.0001 |
| COPD | 562 (5.4) | 61 (7.7) | 623 (5.6) | 0.009 |
| Hemodialysis | 19 (0.2) | 6 (0.8) | 25 (0.2) | 0.004 |
| Diabetes | 1634 (15.8) | 147 (18.5) | 1,781 (16.0) | 0.05 |
| Malignancy | 1481 (14.3) | 130 (16.4) | 1,611 (14.4) | 0.12 |
| NSAIDs | 35 (0.3) | 8 (1.0) | 43 (0.4) | 0.01 |
| Other |  |  |  |  |
| Serum sodium, median (range) | 140(113, 161) | 139(119, 150) | 140(113, 161) | 0.01 |
| Potassium supplement | 3551 (34.3) | 277 (34.9) | 3,828 (34.3) | 0.74 |
| Potassiumchloride (single pill combination +an antihypertensive drug) | 2457 (23.7) | 166 (20.9) | 2,623 (23.5) | 0.08 |
| Potassiumchloride | 1326 (12.8) | 132 (16.6) | 1,458 (13.1) | 0.002 |

K^+^<4,6: Normokalemia

K^+^>4,6: Hyperkalemia

BB:Beta blockers

CCB:Calcium channel blockers

RASi:Renin-angiotensin system inhibitors
MRA: Mineralocorticoid-Receptor-Antagonists

IHD/MI: Ischemic heart disease/Myocardial infarction
COPD: Chronic obstructive pulmonary disease

VT/VF: Ventricular tachycardia/ventricular fibrillation

ICD: Implantable cardioverter defibrillator

NSAIDs: Non-steroidal anti-inflammatory drugs

“Potassium supplement” addressed supplementation as a single pill therapy with an

antihypertensive and as an individual pill.

BB: Beta blockers
CCB: Calcium channel blockers
RASi: Renin-angiotensin system inhibitors

SIADH: Syndrome of inappropriate antidiuretic hormone secretion
