# Supplementary material for: Risk of developing hyperkalemia in patients with hypertension treated with combination antihypertensive therapy – a retrospective register-based study
Source: Hypertens Res. 2024 Oct 31;48(1):378–87. doi: 10.1038/s41440-024-01894-2 (PMC11700848; doi:10.1038/s41440-024-01894-2)
Supplement: Supplementary file 4 — Supplementary Table 4 [file 41440_2024_1894_MOESM4_ESM.docx]

|  | K+ >4.6 mmol/L |
| --- | --- |
| Antihypertensive drug |  |
| BB | 121 (73.8) |
| CCB | 19 (11.6) |
| RASi | 130 (79.3) |
| Diuretics | 15 (9.1) |
| Potassium supplement | 64 (39.0) |

K^+^>4,6: Hyperkalemia

BB:Beta blockers

CCB:Calcium channel blockers

RASi:Renin-angiotensin system inhibitors
MRA: Mineralocorticoid-Receptor-Antagonists

“Potassium supplement” addressed supplementation as a single pill therapy with an

antihypertensive and as an individual pill.

BB: Beta blockers
CCB: Calcium channel blockers
RASi: Renin-angiotensin system inhibitors
