# Supplementary figures and images for: Risk of developing hyperkalemia in patients with hypertension treated with combination antihypertensive therapy – a retrospective register-based study
Source: Hypertens Res. 2024 Oct 31;48(1):378–87. doi: 10.1038/s41440-024-01894-2 (PMC11700848; doi:10.1038/s41440-024-01894-2)

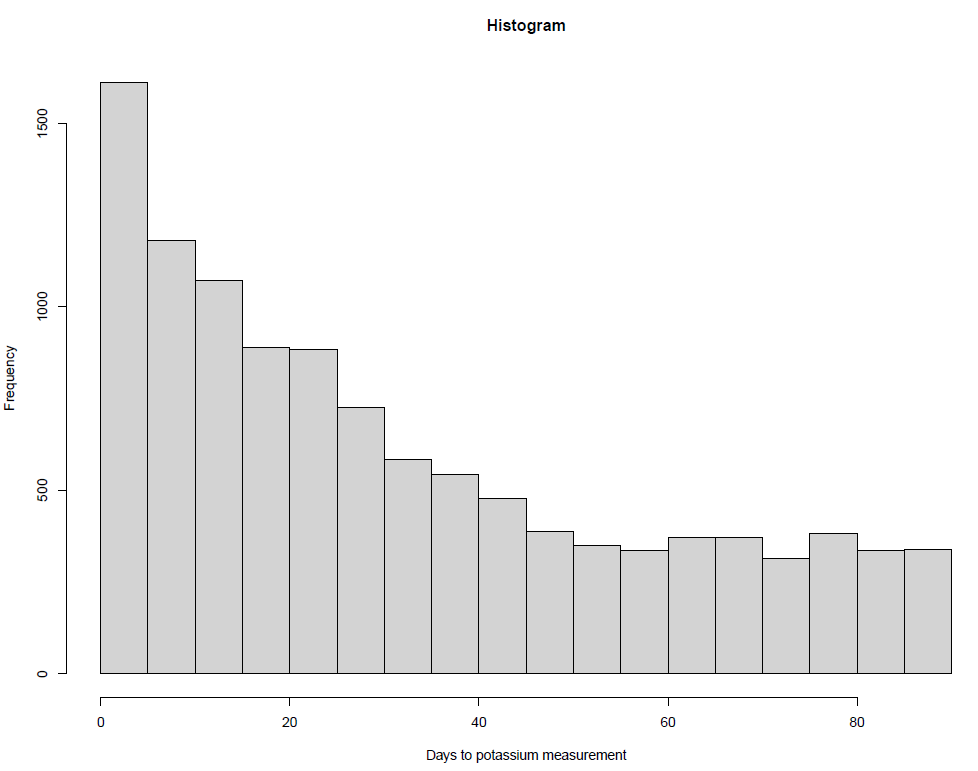

Supplement: Supplementary file 5 — Supplementary Figure 1 [file 41440_2024_1894_MOESM5_ESM.docx]

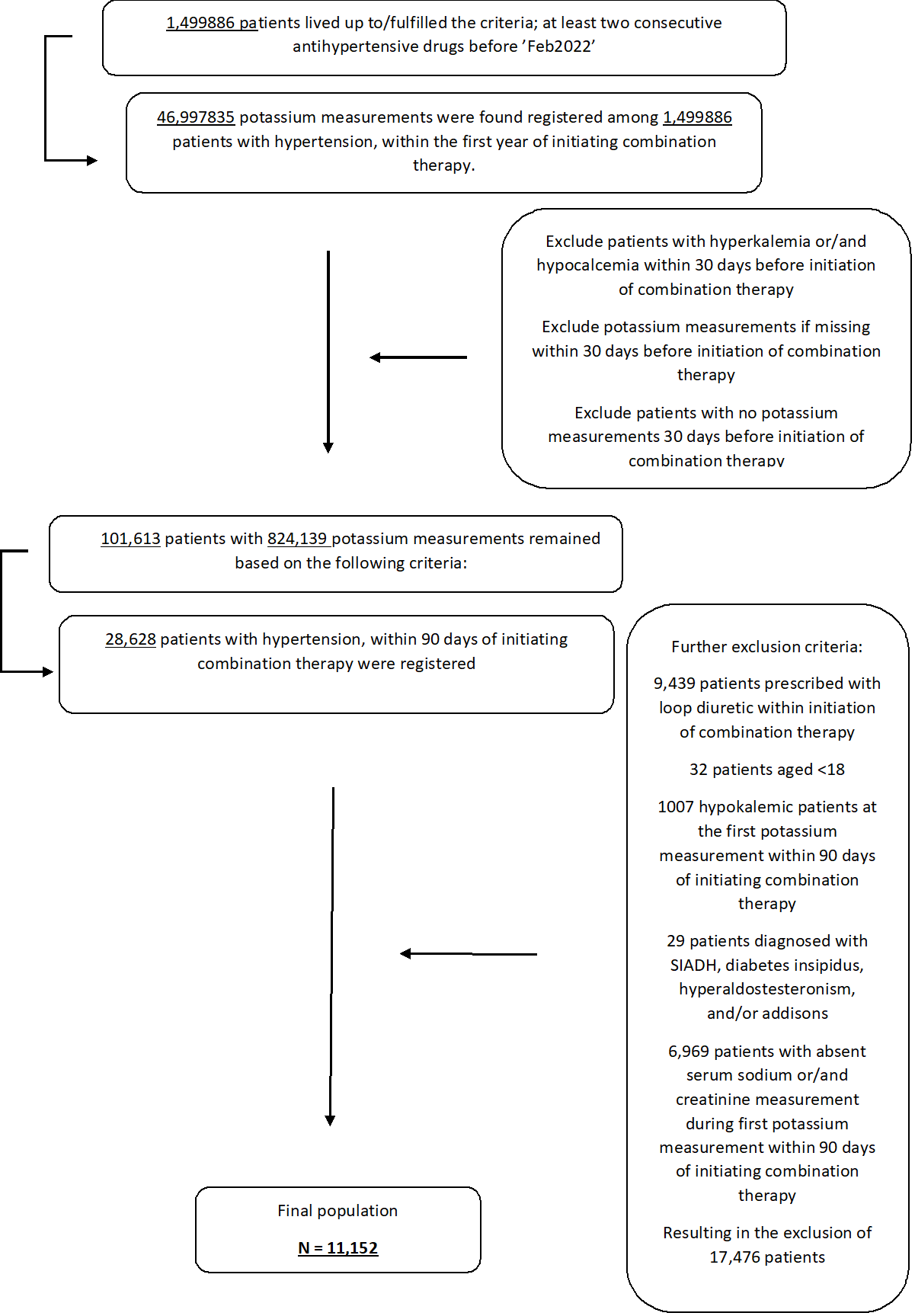

Supplement: Supplementary file 6 — Supplementary Figure 2 [file 41440_2024_1894_MOESM6_ESM.docx]
