# Supplementary material for: Risk of developing hyperkalemia in patients with hypertension treated with combination antihypertensive therapy – a retrospective register-based study
Source: Hypertens Res. 2024 Oct 31;48(1):378–87. doi: 10.1038/s41440-024-01894-2 (PMC11700848; doi:10.1038/s41440-024-01894-2)
Supplement: Supplementary file 7 — Supplementary Figure 3 [file 41440_2024_1894_MOESM7_ESM.docx]

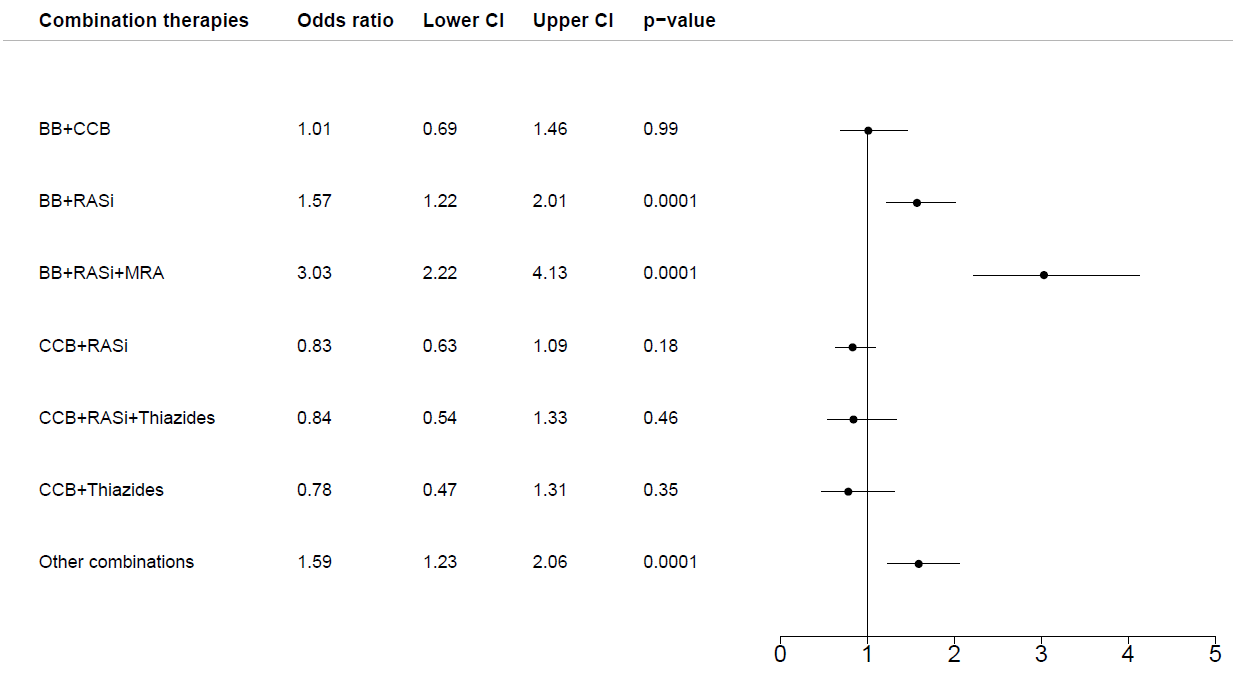


BB:Beta blockers

CCB:Calcium channel blockers

RASi:Renin-angiotensin system inhibitors
MRA: Mineralocorticoid-Receptor-Antagonister
